# From Gynecological Endocrine Disorders to Cardiovascular Risk: Insights from Rat Models

**DOI:** 10.3390/biomedicines13123081

**Published:** 2025-12-13

**Authors:** Csanád Endre Lőrincz, Zoltán Virág, András Nagy, Viktória Kiss, Ákos Tóbiás, Denise Börzsei, Csaba Varga, Renáta Szabó

**Affiliations:** 1 Department of Physiology, Anatomy, and Neuroscience, Faculty of Science and Informatics, University of Szeged, H-6726 Szeged, Hungary; 2 HR-Pharma Ltd., H-6726 Szeged, Hungary

**Keywords:** polycystic ovary syndrome, endometriosis, primary ovarian insufficiency, premature ovarian failure, cardiovascular risk, rat models

## Abstract

Gynecological endocrine disorders, including polycystic ovary syndrome (PCOS), endometriosis as well as primary ovarian insufficiency (POI)/premature ovarian failure (POF), significantly impact women’s reproductive health and overall well-being. While these conditions are primarily driven by disturbances of the hypothalamic–pituitary–gonadal axis, yet growing evidence indicates that oxidative stress plays a crucial role in their development and progression. The combined impact of hormonal imbalance and impaired redox homeostasis contributes to infertility, metabolic dysfunction, and other co-morbidities, such as increased cardiovascular risk. Given that women may live for many years with these chronic conditions, investigating their pathophysiology and associated complications is of particular importance. This narrative review summarizes current knowledge on PCOS, endometriosis, and POI/PMF, emphasizing the contribution of oxidative stress and also highlights the association between these disorders and cardiovascular risk. Furthermore, the utility of rat models is presented to support the advancement of preventive and therapeutic research.

## 1. Introduction

Gynecological disorders such as endometriosis, polycystic ovary syndrome (PCOS), primary ovarian insufficiency (POI), and premature ovarian failure (POF) are prevalent conditions among women of reproductive age [1,2]. Despite their heterogeneity in clinical presentation, they share common features, including the involvement of oxidative stress in their development and progression. 

Oxidative stress, resulting from an imbalance between reactive oxygen species (ROS) production and antioxidant defense mechanisms, plays a key role in disrupting endocrine function, damaging cellular components, and impairing tissue homeostasis [3]. Accumulating evidence suggests that oxidative stress is not only central to the pathophysiology of these disorders but may also act as a mechanistic link between reproductive dysfunction and systemic complications, particularly affecting cardiovascular health [4,5,6,7]. A growing body of research indicates that women with gynecological endocrine disorders are at increased risk of developing cardiovascular complications, including hypertension, atherosclerosis, endothelial dysfunction, and heart failure [8]. These associations underscore the need to understand shared molecular pathways, including ROS-mediated endothelial dysfunction, which may underpin both gynecologic and cardiovascular pathologies.

Rodent models, particularly rat models, have been essential in advancing our understanding of the pathophysiological mechanisms underlying these conditions [9]. Through experimental manipulation, such models enable controlled investigations into hormonal signaling, oxidative stress responses, and systemic outcomes. Moreover, they provide a valuable approach for testing potential therapeutic strategies aimed at mitigating not only reproductive symptoms but also long-term cardiovascular risks.

Therefore, our narrative review aims to highlight the role of sexual hormone changes and oxidative stress in the context of endometriosis, PCOS, POI, and POF, with a focus on the relevance of animal models—particularly those chemically or pharmacologically induced—in research, and the associated cardiovascular risks. Animal models can provide a valuable approach for testing potential therapeutic strategies aimed at mitigating not only reproductive symptoms but also long-term cardiovascular risks.

## 2. Disorders of Ovarian Function

### 2.1. Primary Ovarian Insufficiency (POI) and Premature Ovarian Failure (POF)

#### 2.1.1. Hormonal Changes and Oxidative Stress in POI and POF

Primary ovarian insufficiency (POI) was previously referred to as premature ovarian failure; however, this terminology has receded into the background as the degree of ovarian impairment may fluctuate over time [10]. Therefore, these two fertility-damaging conditions are now separated. POI is defined as depletion or dysfunction of ovarian follicles with intermittent ovulation before the age of 40 [11], while premature ovarian failure (POF) is characterized by the cessation of ovarian function.

Ovarian depletion and dysfunction result that granulosa cell, which are the primary source of estrogen, disappear; thus, serum estrogen level decreases. Consequently, the endometrium does not thicken and the absence of corpus luteum inhibits progesterone secretion, which eventually leads to amenorrhea. Hormonal imbalance in women with POI is associated with low estradiol (E2) and elevated FSH level. Previously, various elevated FSH thresholds were used as diagnostic criteria; however, cases with lower serum FSH levels have also been reported [12]. Currently, the most widely accepted diagnostic criteria are those recommended by the European Society of Human Reproduction and Embryology (ESHRE) Guideline Development Group for premature ovarian insufficiency (POI). According to these guidelines, POI is defined by oligo- or amenorrhea lasting at least four months, accompanied by elevated serum FSH levels > 25 mIU/mL on two separate occasions, obtained at least four weeks apart [12]. Similar to POI, POF is characterized by elevated FSH levels, as well as reduced E2 and anti-Müllerian hormone (AMH) levels, while ultrasound examination typically reveals an absence of ovarian follicles and an atrophic, shrunken uterus with a thin endometrium [13]. 

**Figure 1:**
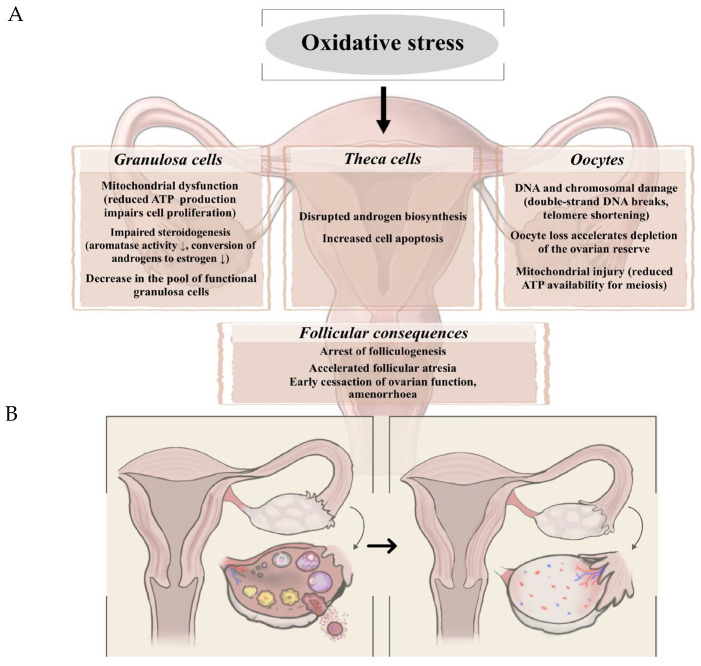
(**A**) Mechanistic explanation of the relationship between oxidative stress and POI/POF. (**B**) Schematic representation of ovarian changes associated with POI/POF.

A large number of studies suggest that the development of POI is associated with oxidative stress and inflammation. Excessive ovarian accumulation of ROS accelerates the aging of ovarian cells and reduces ovarian function [14]. Ovarian aging leads to a reduction in the granulosa cell’s ability to counteract the harmful effects of oxidative stress, which leads to the reduction in the quality and number of follicles and ultimately to POI. In a previous study, Kakinuma et al. brought to light that reactive oxygen metabolites and antioxidant potential are closely linked to the number and development of residual oocytes and follicles. They concluded that oxidase stress index was significantly higher in the POI group compared to the control (healthy) participants [15]. Both POI and POF are closely related to mitochondrial dysfunction and a consequent ROS production, which eventually culminate in lack of histone protection and antioxidant defense. Both POI and POF have been closely linked to mitochondrial dysfunction and the resulting overproduction of reactive oxygen species (ROS), which ultimately leads to compromised histone protection and impaired antioxidant defense mechanisms. It has been proposed that ovarian aging is exacerbated by the accumulation of mitochondrial impairments, including mitochondrial DNA (mtDNA) mutations, imbalances in mitochondrial dynamics (fusion and fission), and defects in the electron transport chain [16]. This relationship appears to be bidirectional, as the aging process itself further deteriorates mitochondrial quality and function [17,18]. Beside oxidative stress-related findings, recent studies have shown that inflammatory aging is closely related to these endocrine pathologies [19]. It is known that aging induces a low-grade inflammation in the body with a negative effect on fertility and contributes to POI and POF. Kunicki et al. summarized that serum concentration of pro-inflammatory cytokines such as IL-6, IL-8 and TNF-α increased, while anti-inflammatory cytokines, such as IL-10 decreased [20]. Figure 1 summarizes the mechanistic link between oxidative stress and POI/POF and the ovarian changes.

#### 2.1.2. POI, POF, and Cardiovascular Disorders

A growing body of evidence demonstrates a strong relationship between cardiovascular health and the levels of endogenous sex hormones. Among sexual steroids, estrogen hormone has a major impact on cardiovascular homeostasis through its pleiotropic regulatory features [21]. Under physiological condition, estrogen possesses antioxidants, antiapoptotic and anti-hypertensive properties; however, decline in ovarian function and reduction in estrogen level can be associated with adverse cardiac effects. If the balance between antioxidant defense mechanisms and ROS disrupts, ROS clearance decreases, which leads to oxidative stress and a consequent destruction of cardiac homeostasis. Excessive ROS production in estrogen-deficient condition is considered one of the most important pathogenic mechanisms of atherosclerosis, myocardial infarction, hypertrophy and heart failure. In the vasculature, nicotinamide adenine dinucleotide phosphate (NADPH) oxidase 4 (NOX4) isoform is the main source of the ROS. During oxidative stress, reactive oxygen species (ROS) impair endothelial function by disrupting nitric oxide (NO) signaling. Normally, NO diffuses into vascular smooth muscle cells, activates soluble guanylyl cyclase, and increases cyclic guanosine monophosphate (cGMP), promoting vasodilation. NO also inhibits leukocyte adhesion and platelet aggregation, thereby protecting the endothelium and limiting atherogenesis. However, disruption of this tightly regulated mechanisms can promote the formation of the fibrous plaque, which is the hallmark of atherogenesis [22]. In this regard, nitric oxide (•NO) can be antagonized by superoxide (O_2_^−^•) radicals, leading to the formation of the cytotoxic oxidant peroxynitrite (ONOO^−^), which serves as a marker of endothelial dysfunction [23,24].

Previous findings highlight the importance of reduced level of free thiols in the incidence and progression of cardiovascular diseases. Free thiols are considered a potent biomarkers of redox status due to their ROS-scavenging properties. In addition to the free thiols that are mostly incorporated into circulating cysteine-based proteins, low-molecular-weight free thiol, such as homocysteine, glutathione, and coenzyme, form a dynamic system that mediates kinetically regulated redox exchange processes both inside and outside cells [25,26]. Therefore, disruption in this regulatory circuits and reduction in free thiol level lead to shifts in antioxidant capacity and a consequent increase in cardiovascular risk. Previous findings show that higher serum levels of free thiols are associated with favorable outcomes in patients with chronic heart failure [27] and in pre-postmenopausal women [28]. Furthermore, Isik et al. demonstrated that thiols serve as a predictive biomarker in patients with POF [29]. In a recent study, Lira-Silva et al. provided evidence that estrogenic degradation metabolites can be associated with an increased cardiovascular risk in menopausal women. They found that quinones derived from estradiol hydroxylation can produce ROS, such as 8-OHdG, and directly damage DNA. In estrogen-deficient conditions, this oxidative damage cannot be effectively controlled due to estrogen loss and lower levels of antioxidant enzymes [30]. Although estrogen loss is an independent risk factor for cardiovascular diseases, the accompanying metabolic changes, such as obesity, insulin resistance, and dyslipidemia, may exacerbate cardiac outcomes in women with estrogen-deficient endocrine disorders [31,32]. The direct link between POI/POF and cardiovascular diseases has been less studied compared to other reproductive issues such as menopause. Yorgun et al. showed that young women with POF exhibit impaired endothelial function, reduced circulating endothelial progenitor cells (EPCs), increased carotid intima–media thickness (CIMT), and diastolic dysfunction. The study also identified a direct link between reduced EPC levels and POF, and suggested that elevated CIMT in this young cohort may predict clinically relevant carotid abnormalities later in life [33].

#### 2.1.3. Experimental Rodent Models of POI and POF

Based on the broad range and complex etiology of POI and POF, various animal models have been developed to mimic the disease. Different animal model methods have their respective advantages and disadvantages; therefore, selecting the optimal model for studying the underlying molecular mechanisms remains a challenge.

Galactose (GAL)-induced animal model mimics the physiological aging characteristics of patients with ovarian dysfunction. Galactose exposure targets follicular development. While primordial and primary follicles are targeted by galactose toxicity during prenatal exposure and the pre-antral and antral follicles during postnatal exposure. GAL becomes ovotoxic when excessive amounts are metabolized into galactose-1-phosphate and galactitol, which accumulate within ovarian cells. This leads to osmotic swelling, mitochondrial dysfunction, and oxidative stress, damaging granulosa and theca cells, impairing estrogen production, arresting follicle maturation, and ultimately resulting in follicle depletion and premature ovarian failure. This ovotoxic effect can be used to induce appropriate POI animal models with sufficient doses, proper onset time, and duration of prenatal exposure. The clinical relevance of this model is that the most common long-term complication of galactosemia is POI in at least 80% of all girls and women [34]. In a pilot study, Wistar rats were fed with galactose-enriched diet in different stages during pregnancy and were divided into 4 groups. The study revealed no significant differences in the birth weight of the offspring; however, the ovarian index of the GAL-fed offspring was significantly compared to control offspring [35].

Several POI and POF models have been developed and can be broadly categorized into chemotherapy-, radiation-, stress-, and autoimmune-induced models. The pathophysiology of chemotherapy-associated POI and POF is primarily related to the genotoxic effects of these agents, which induce apoptosis in ovarian follicles and impair ovarian vascularization [36]. Among chemotherapy drugs, cisplatin belongs to alkylating agents, which forms cross-links between DNA chains and inhibits follicle DNA transcription and synthesis [36] as well as increases cellular oxidative stress by hydrolyzing the drug cisplatin in the cell and fusing it with GSH, cysteine (L(+)-Cysteine), and other proteins leading to the depletion of antioxidant reserves in the cytoplasm [13]. In a recent study, Du et al. underpin a strong link between cisplatin induced POI and ferroptosis [37]. Cisplatin was also used by Dinc et al., and they confirmed cisplatin-associated MDA increase in the ovarian tissue. Histopathological analyses revealed structural disruption of ovarian tissue and degeneration of developing follicles, characterized by necrotic cell accumulation within and around follicular structures, extensive tissue and corpus luteum hemorrhage, and localized interstitial edema [38]. Another chemotherapy drug, cyclophosphamide combined with busulfan, was used by Tang et al. [39]. Cyclophosphamide exerts its effects through a combination of direct DNA damage to ovarian follicles and accelerated follicle depletion mediated by oxidative stress and apoptotic pathways, whereas busulfan is a bifunctional alkylating agent that inhibits DNA replication and transcription, primarily in dividing cells (including granulosa cells and oocytes), leading to DNA damage and ultimately apoptosis. Tang et al. demonstrated that the combined administration of cyclophosphamide and busulfan establishes a more reliable POI model while minimizing potential adverse effects. Accordingly, this rat model provides a stable and reproducible platform for investigating chemotherapy-induced ovarian damage. The use of cyclophosphamide alone to induce POI has been documented by Yamchi et al. [40] and Fan et al. [41]. In both studies, POI was confirmed through histological evaluation and measurement of hormone levels, which revealed decreased E2 and AMH levels.

POI can also be reliably induced by 4-vinylcyclohexene dioxide (VCD) [42]. VCD is a widely used industrial chemical and environmental pollutant, primarily utilized in the production of rubber products, pesticides, plasticizers, and other everyday consumer materials. Its ovotoxicity in rats has been firstly reported by Flaws et al., and proved that VCDs can distrupt the viability of granulosa cells, the companions of the oocytes by inhibiting their proliferation, causing DNA damage, and increasing apoptosis [43].

A novel approach in POI induction was used by He et al. using dynamic intensity modulated radiation therapy. POI was confirmed by reduced ovarian weight, altered hormone levels (decreased serum luteinizing hormone (LH), follicle-stimulating hormone (FSH), and E2), and a disrupted estrous cycle [44].

Among autoimmune-induced models, Wang et al. demonstrated that ovarian antigen injection successfully induced POF, causing ovarian damage, decreased granulosa cells, and significantly lower serum levels of E2 and AMH. Further observations revealed that the follicles displayed an irregular, non-rounded shape [45]. 

Studies have shown that psychological stress, including chronic anxiety, sadness, fear, and other negative emotions, can contribute to POF by altering the function of the hypothalamic–pituitary–adrenal axis. Wang et al. successfully induced POF in Sprague Dawley rats using acousto-optical–electric stimulation and confirmed the development of POF with hormone measurements [46]. A similar, albeit slightly different method was used in a pilot study by Fu et al., who applied a so-called ‘chronic unpredictable mild stress’ or CUMS method. CUMS is a widely used approach for modeling depression, exposing animals to a range of chronic mild stressors, such as restraint, fasting, forced swimming, and altered light–dark cycle, which elicit variable and unpredictable psychological stress responses compared with single-factor models [47]. The CUMS model has been shown to induce psychological stress and disrupt ovarian function in rats; however, further research is needed to determine its effectiveness in inducing POF in experimental animals.

Although Tripterygium glycoside exhibits antibacterial, immunosuppressive, and antitumor properties, it is also associated with reproductive toxicity, which may contribute to the development of POF. The primary mechanism underlying its toxicity appears to be its pro-apoptotic effects, followed by the induction of inflammation and oxidative stress [48]. In terms of its impact on hormonal regulation, its mechanism of action involves a synergistic interaction with LH and FSH, which in turn influences E2 secretion. However, Tripterygium glycoside has been shown to reduce serum levels of E2 and AMH while increasing FSH and LH, thereby disrupting the reproductive endocrine balance [48]. As a result, follicle count and E2 levels decline, leading to impaired follicular development and oocyte maturation [49]. In a recent study, Han et al. established a POF model using glycosides from *Tripterygium wilfordii*, a plant used in traditional Chinese medicine. Sprague Dawley rats were administered tripterygium glycosides *intragastrically* at a dose of 40 mg/kg/day for ten weeks. POF was confirmed by histological analysis of ovarian morphology and by assessment of serum hormone levels [50]. Table 1 presents the rat models of POI/POF.

### 2.2. Polycystic Ovary Syndrome (PCOS)

#### 2.2.1. Hormonal Changes and Oxidative Stress in PCOS

**Figure 2:**
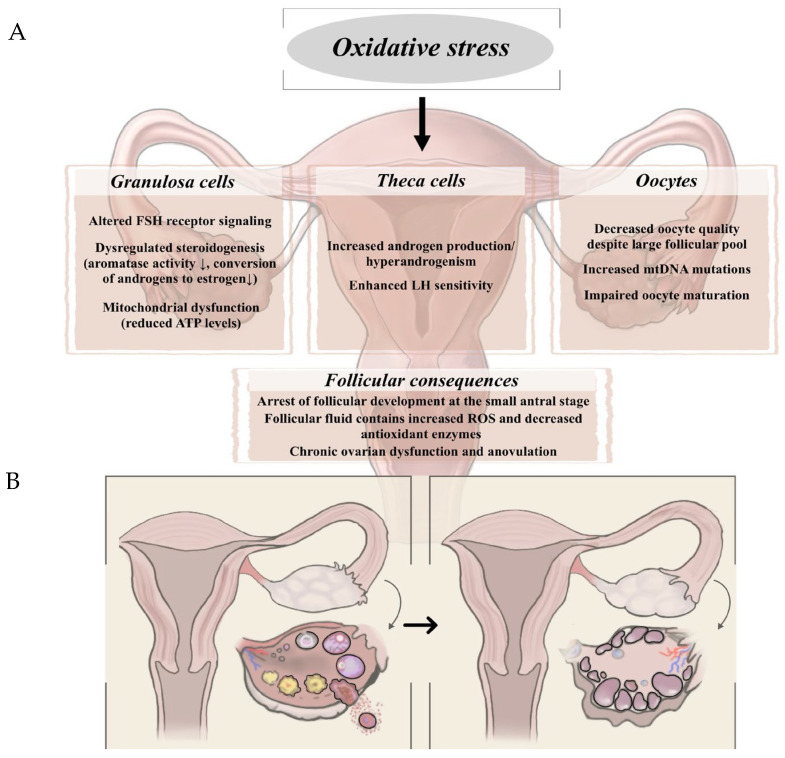
(**A**) Mechanistic explanation of the relationship between oxidative stress and PCOS. (**B**) Schematic representation of ovarian changes associated with PCOS.

Polycystic ovary syndrome (PCOS) is a heterogeneous endocrine disorder characterized by reproductive and metabolic dysfunctions, with an estimated prevalence of 6–13% in reproductive-age women worldwide [1]. Diagnosis is based on the Rotterdam criteria, which require the presence of ovulatory dysfunction, hyperandrogenism, and polycystic ovarian morphology [58]. Although weight gain and obesity are common features in the development and progression of PCOS, lean phenotypes are increasingly recognized and may exhibit distinct hormonal profiles. Obese PCOS patients typically exhibit insulin resistance and metabolic syndrome components [59,60], whereas lean patients often show higher LH levels and an elevated LH/FSH ratio [61]. One of the hallmark features within disrupted hypothalamic–pituitary–ovarian (HPO) axis is an elevated LH to FSH ratio, which contributes to disrupted folliculogenesis and excess androgen production by ovarian theca cells [62]. On the other hand, insufficient FSH activity impairs follicular development, leading to anovulation and the formation of polycystic ovarian morphology [63]. Additionally, altered estrogen dynamics, notably an increased estrone-to-estradiol ratio, further disturb hypothalamic and pituitary feedback mechanisms [64]. Figure 2 summarizes the mechanistic link between oxidative stress and PCOS and its consequences in the ovaries.

A growing body of research underscores the pivotal role of oxidative stress in the pathogenesis of PCOS, with key oxidative markers offering valuable insights into its molecular mechanisms. Elevated level of MDA is consistently observed in women with PCOS. Jeelani et al. demonstrated that serum MDA levels in PCOS patients were significantly higher than healthy controls [65]. In addition to lipid peroxidation, 8-OHdG serves as a prominent biomarker for oxidative DNA damage. Elevated 8-OHdG levels reflect the significant DNA damage induced by ROS in PCOS patients. This damage is particularly evident in ovarian cells, impairing normal follicular development and steroidogenesis. The accumulation of 8-OHdG also suggests a long-term impact on cellular integrity, potentially leading to chronic inflammation and further metabolic dysfunction, and possibly contributing to the deterioration of ovarian function over time [66]. Novel studies and emerging evidence increasingly suggest a link between mitochondrial dysfunction and PCOS [67]. Several features of PCOS negatively affect mitochondrial homeostasis, including androgen excess and insulin resistance. Hyperandrogenism disrupts mitochondrial electron transport, lowers ATP production, and impairs mitochondrial biogenesis [68], whereas insulin resistance perturbs cellular metabolism and elevates oxidative stress, further compromising mitochondrial function [69]. 

#### 2.2.2. PCOS and Cardiovascular Disorders

PCOS is not only related to reproductive health, but also includes a number of systemic symptoms, making it a multifaceted endocrine disorder. PCOS can lead to cardiometabolic abnormalities, such as atherogenic dyslipidemia, insulin resistance, glucose intolerance, hypertension or metabolic syndrome, which can have serious effects on cardiovascular health [70]. Insulin has pleiotropic effects, which serves as a key regulator of glucose regulation, lipogenesis as well as promotes steroidogenesis in steroidogenic tissues. Consequently, disturbances in insulin homeostasis can be strongly associated with obesity and dyslipidemia; moreover, insulin resistance (IR) results in elevated androgen secretion and decreases the synthesis of sexual hormone binding protein (SHBG) [71]. Furthermore, elevated androgen level positively correlates with LH levels, thereby impairing the negative feedback mechanism, which eventually leads to PCOS with increased cardiometabolic risk [72]. In PCOS, insulin resistance and hyperinsulinemia impair cardiovascular homeostasis by promoting vascular smooth muscle hypertrophy and reduced compliance, disrupting endothelium-dependent vasodilation, and activating the renin–angiotensin–aldosterone system with sodium retention, ultimately contributing to chronic hypertension. [73]. Additionally, it is important to note that among women of reproductive age, the prevalence of obesity and hypertension is higher in those with PCOS compared to healthy controls, independent of BMI [74].

At molecular level, metabolic disruption caused by PCOS also has a significant impact on ROS level, which leads to an increased oxidative stress and cellular damage. As mentioned previously, excessive ROS induce DNA damage and/or cell apoptosis, thereby disrupting gene expression and impairing the immune response [75,76].

Although the exact mechanisms driving ROS production in PCOS remain unclear, several factors contribute to its increased levels in this condition [75]. Hyperglycemia and insulin resistance are key drivers of oxidative stress, largely mediated by p47(phox) activation and subsequent TNF-α release [77]. NF-κB further exacerbates oxidative stress by activating NADPH oxidase, increasing ROS production, and sustaining inflammation. [78]. Elevated oxidative stress can induce hyperandrogenemia, sensitizing leukocytes to hyperglycemia and further amplifying ROS in a vicious cycle. In women of reproductive age, hyperandrogenism is associated with subclinical markers of atherosclerotic cardiovascular disease, including arterial stiffness, increased carotid intima–media thickness, coronary artery calcification, and endothelial dysfunction [79]. Endothelial dysfunction can be associated with elevated peroxynitrite level, which is formed through the reaction of NO with superoxide anion. A large number of studies underpin that obesity and insulin resistance are contributors to the peroxynitrite-driven cardiovascular risk [80]. Sprung et al. reported endothelial dysfunction in women with PCOS, even in young and non-obese individuals [81]. A meta-analysis by Meng et al. further demonstrated that decreased serum or plasma nitrite levels are associated with PCOS, suggesting that endothelial dysfunction may contribute to its pathogenesis [82].

The adverse cardiovascular effects of PCOS are also mediated by advanced oxidation protein products (AOPPs), which were significantly elevated in PCOS patients, as shown by Kaya et al. [83]. AOPPs are considered novel markers of oxidant-mediated protein damage. Although AOPPs are byproducts of chronic oxidative stress, they can further exacerbate oxidative damage and induce pathological cellular processes, including apoptosis, autophagy, and pyroptosis [84]. Altered AOPP levels have been reported in the peritoneal and follicular fluids of patients with endometriosis, with follicular fluid AOPP concentrations negatively correlating with blastocyst formation rates [85]. In women with PCOS, elevated AOPP levels are linked to increased atherosclerotic cardiovascular risk alongside established factors such as obesity, insulin resistance, homocysteine, MDA, and CRP. A positive correlation between serum AOPP and carotid intima–media thickness further supports their association with early subclinical atherosclerosis [86].

#### 2.2.3. Experimental Rodent Models of PCOS

To mimic human PCOS, several animal models were developed and used over the years. Due to the complexity of PCOS and its myriad symptoms and forms, various animal models were developed. Hyperandrogenism being one of the pillars of PCOS, most rodent models involve direct androgen treatments, either prenatally or postnatally administered. One of the most common model of androgen-treated models is dehydroepiandrosterone (DHEA)-induced PCOS. DHEA is a metabolic intermediate in testosterone biosynthesis and is converted into testosterone in theca cells within the ovaries. Elevated androgen level suppresses aromatase activity in the granulosa cells, reducing their capacity to convert testosterone into E2, which leads to decreased E2 production and impaired follicular maturation. DHEA-treated animal models recapitulate key features of human PCOS, with prepubertal female rats developing ovarian cysts and showing acyclicity or anovulation. At the end of the treatment period, PCOS condition were verified by serum hormone tests, analysis of body fat distribution as well as histological analysis on the reproductive organs [87]. Although DHEA-induced pre-pubertal rat model for PCOS has been widely used, the model exhibits weaknesses such as decreased ovary weight. To combat this very issue, Kim et al. used an innovative technique in post-pubertal rats. Forty-two-day-old SD rats were injected with DHEA dissolved in sesame oil (60 mg/kg body weight), up to 20–30 days. Whereas in pre-pubescent rats showed irregular PCOS phenotypes (such as increased body weight, but decreased ovarian weight), the post-pubertal rats exhibited increased ovarian size, increased uterine weight and size. Post-pubertal exposure to excessive DHEA improved reproducibility, PCOS induction ratio, ovarian cyst formation, and uterine malformation [88]. 

Since DHT is a non-aromatizable androgen and cannot be converted to E2 by aromatase, its exogenous administration establishes and maintains a hyperandrogenic state through both direct and indirect mechanisms [89]. DHT-induced PCOS phenotype can be analyzed in DHT-treated prenatal or postnatal animals without taking into account the effects of estrogen converted from androgens [90]. The most widespread model used for prenatal DHT-treated rats is 3 mg of DHT daily from gestational day 16 to 19. Offspring prenatally exposed to DHT served as PCOS models, displaying irregular estrous cycles and PCO-like ovarian morphology. Prenatal DHT also increased LH levels and kisspeptin expression in the ARC, without affecting body weight. For postnatal exposure, rats received subcutaneous DHT pellets starting on postnatal day 21 [90,91]. Postnatal DHT-treated rats exhibit similarities to the human PCOS phenotype; however, some characteristics differ. Notably, LH levels remain unchanged and ovarian volume is reduced in this rat model. On the positive side, metabolic parameters, including insulin resistance and adiposity, are comparable to those observed in human PCOS. Therefore, this model is suitable for investigating the metabolic aspects of PCOS [90]. PCOS was also successfully induced in the DHT-rodent model used by Gao et al., who used an injection of 5α- DHT at dose of 7.5 mg [92]. In another model by Kamada et al., PCOS was induced by DHT–oil (a solution of 80% peanut oil and 20% ethanol) combination starting on postnatal day 26. Their modified rat model verifies reproduced the reproductive and metabolic features of PCOS without any atrophic changes in the ovaries or uterus, which are observed in conventional DHT-induced PCOS model animals [93].

Aromatase is an enzyme responsible for converting testosterone and androstenedione to estradiol (E2) and estrone. Letrozole, a nonsteroidal aromatase inhibitor, blocks this conversion, leading to increased androgen levels [94]. Letrozole via postnatal administration has been used to create animal models of PCOS [90,95,96]. Letrozole-induced PCOS rats exhibit acyclicity, cystic ovarian morphology corresponding to human PCOS, and elevated serum LH levels [90]. 

There are also testosterone-induced PCOS models, such as the one used by Tehrani et al., who injected ten pregnant rats with 5 mg free testosterone on gestational day 20. The prenatally androgenized offspring had irregular estrous cycles and an increased number of preantral and antral follicles in the ovaries [97]. Prenatal exposure to a single dose of testosterone during the critical period of fetal development affects the hypothalamic–pituitary–ovarian axis, leading to an ovarian morphology and hormonal profile in adulthood that closely resemble those observed in PCOS subjects. This approach can thus facilitate the development of a functional rat model of PCOS in adulthood, with minimal morphological disruptions in the reproductive system [97]. Another model was applied by Beloosesky et al., who used Wistar rats 21 days of age, injecting them daily with testosterone propionate for up to 35 days to achieve PCOS-phenotype in the rats. Furthermore, the observation of normal or low glucose levels alongside high insulin levels in testosterone-treated rats suggests that elevated androgens may contribute to insulin resistance in this experimental PCOS model [98].

Another rat model used for PCOS is the progesterone receptor antagonist model by using RU486 or mifepristone. In a RU486-induced PCOS model [99], follicular growth arrest and an increased rate of follicular atresia were observed, along with elevated serum LH concentration. 

Estrogen can also be used to manifest PCOS-like symptoms in rats, as demonstrated by Brawer et al. [100] or more recently by Venegas et al. [101], where young Wistar rats were injected with estradiol valerate (EV). EV administration in rats induces a prolonged high-estrogen state that suppresses FSH and alters GnRH pulses, resulting in an elevated LH/FSH ratio. This disrupts follicular maturation, causes follicular arrest, and produces polycystic ovarian morphology with hyperandrogenism. EV-induced PCOS model rats exhibited cystic ovarian morphology; however, ovary weight was decreased by administration of a single dose of 2 mg/body. A drawback is that estrogen models are limited by the lack of endocrine and metabolic features associated with human PCOS [101]. Table 2 summarizes the rat models of PCOS.

## 3. Endometriosis

### 3.1. Hormonal Changes and Oxidative Stress in Endometriosis

Endometriosis is one of the most common forms of reproductive ailments in the world, affecting about 18% of women of reproductive age [107]. Endometriosis is a chronic systemic inflammatory disorder in which endometrial-like tissue is found outside the uterine cavity. While the ovaries and pelvic peritoneum are the primary sites of involvement, lesions can also appear in the fallopian tubes, abdominal wall, cervix, bladder, and vagina [108]. 

Endometriosis can be associated with hormonal imbalance, which ultimately disrupts reproductive function. In a normal menstrual cycle of healthy, fertile women, the pituitary gland secretes LH and FSH to stimulate the growth of ovarian follicles. These follicles, through positive and negative feedback mechanisms, subsequently induce an LH surge that signals ovulation at the optimal time. Consequently, women with endometriosis suffer from abnormal LH cycle and prolonged follicular phase, which ultimately results in lower LH and increased FSH levels [109]. A disturbance in the LH:FSH balance also brings with it further complications, such as lowered androgen and increased estrogen levels. Endometriosis is therefore widely regarded as an estrogen-dependent disorder. Endometriotic lesions can express steroid hormone related genes, including aromatase, allowing for local de novo production of estradiol (E2). Dysregulation of estrogen-metabolizing enzymes increases estrogen synthesis and decreases inactivation, leading to locally elevated estrogen levels that promote further proliferation of ectopic endometrial tissue [110]. Increased estrogen levels contribute to the survival and proliferation of endometrial lesions and the production of inflammatory cytokines, leading to localized chronic inflammation, pain, and discomfort, all of which are hallmark symptoms of endometriosis.

**Figure 3:**
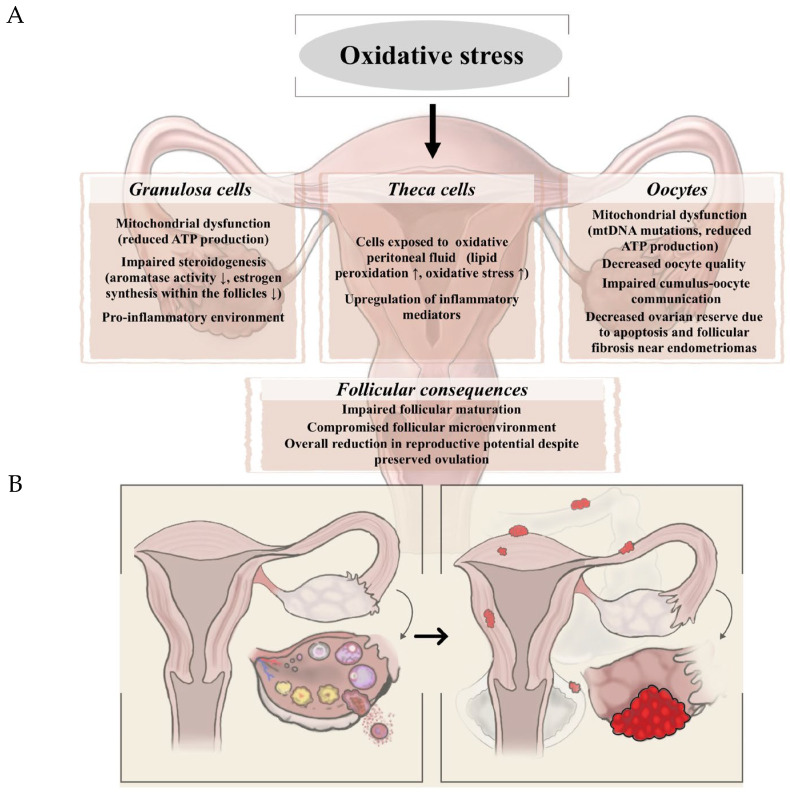
(**A**) Mechanistic explanation of the relationship between oxidative stress and endometriosis. (**B**) Schematic representation of the appearance of endometrial lesions.

Emerging evidence highlights oxidative stress as a central player in the pathophysiology of endometriosis. Increased ROS level and impaired antioxidant defenses have been consistently observed in different samples and tissues, including peritoneal and follicular fluid, endometrial tissue and serum of women with endometriosis. Polak et al. demonstrated that significantly higher concentrations of 8-hydroxy-2’-deoxyguanosine (8-OHdG) and 8-isoprostane concentrations in peritoneal fluid were correlated with advanced stages of endometriosis [111]. Further oxidative stress markers, such as malondialdehyde (MDA) [112], advanced oxidation protein products (AOPP) [85], and NO [113], are associated with disease severity. While ROS levels are elevated in patients with endometriosis, antioxidant enzyme levels are often reduced in affected individuals [114]. Beside oxidative stress, several biological factors can exacerbate the progression of endometriosis, including hormonal imbalance, inflammation, and enhanced angiogenesis. Among these, hyperprolactinemia has emerged as a potential contributor to disease severity, both through its endocrine effects and its interactions with oxidative and angiogenic pathways. Because endometriosis and elevated prolactin levels are independently associated with infertility early hypotheses proposed that hyperprolactinemia might contribute directly to infertility in women with endometriosis [115]. Mirabi et al. reported that infertile women with advanced endometriosis exhibit significantly higher serum prolactin levels than infertile women without the disease, suggesting that prolactin may serve as both a contributing factor and a potential biomarker for endometriosis [116]. Importantly, prolactin exerts immunomodulatory and pro-angiogenic effects, including the stimulation of vascular endothelial growth factor (VEGF) expression and the amplification of inflammatory cytokine production [117]. These processes are closely linked to ROS production, as oxidative stress enhances angiogenesis and inflammation within the peritoneal microenvironment. Elevated prolactin may therefore indirectly potentiate oxidative stress-driven pathways, further promoting lesion survival and disease progression. Lastly, it is important to highlight that metabolic factors, such as insulin resistance and obesity also contribute to an increased ROS production [118]. Figure 3 summarizes the mechanistic link between oxidative stress and endometriosis as well as presents the appearance of the endometrial lesions.

### 3.2. Endometriosis and Cardiovascular Disorders

Among the various complications associated with endometriosis, its connection to the cardiovascular system is frequently emphasized. As shown by Marchandot et al., women with endometriosis have a 1.4× likelihood of developing ischemic heart disease, a 1.19× increased risk for cerebrovascular disease [119], and a strong relationship with atherosclerosis was also proved. While the exact underlying molecular mechanisms between endometriosis and cardio-and cerebrovascular diseases are not fully elucidated, previous studies verified that chronic inflammation, increased oxidative stress, and a consequent endothelial dysfunction are the hallmarks of the diseases [120,121]. These results were confirmed by a large population-based retrospective cohort study including a total of 279,759 women aged 16–50 years. The study demonstrated a definitive association between cardiovascular diseases (CVDs) and endometriosis [122]. Oxidative stress-induced ROS accumulation mediates endothelial dysfunction and vascular abnormalities by disrupting the vasoprotective NO signaling pathway [123]. Under physiological conditions, nitric oxide (NO) functions as a crucial cytoprotective molecule with potent vasodilatory properties. NO inhibits platelet activation and neutrophil adhesion, and it exerts protective effects against ischemia–reperfusion injury and heart failure [124]. Disruption of the NO signaling pathway leads to endothelial dysfunction, contributing to the development of hypertension and atherosclerosis. An association between increased ROS levels and the severity of atherosclerotic lesions in human coronary arteries was demonstrated by Sorescu et al. [125]. Another diagnostic marker for endometriosis, as shown by Verit et al., is the activity of the Serum paraoxonase-1 (PON-1) and lipid hydropersoxide (LOOH), PON-1 being a high-density lipoprotein (HDL)-associated enzyme that prevents oxidative modification of low-density lipoprotein (LDL) [126]. Through a careful study Verit et al. found that women who suffer from moderate to severe endometriosis had significantly lower PON-1 activity and significantly higher LOOH levels. Reduced serum PON-1 activity and increased LOOH might contribute to the increased susceptibility for the development of atherosclerosis.

More recently, Smyk et al. [127] reported a higher prevalence of endothelial dysfunction in patients with endometriosis. In the relationship among endometriosis, oxidative stress, and cardiovascular diseases, the role of mitochondrial DNA (mtDNA) need to be highlighted. Due to its close proximity to the electron transport chain and limited repair capacity, mtDNA is particularly vulnerable to ROS-induced damage. Studies in myocardial infarction models have demonstrated that elevated mitochondrial ROS leads to decreased mtDNA transcription and integrity, promoting mitochondrial dysfunction [128]. This cascade contributes to pathological processes such as cardiomyocyte hypertrophy, fibrosis, and ultimately heart failure [129]. In a recent study, Rafi et al. found that inflammation in endometriosis causes a hypercoagulable status, which leads to the development of atherosclerosis and other cardiac complications, including coronary heart disease, cerebrovascular disease, microvascular thrombosis and acute coronary syndromes [130]. Continuing the list of the relationship of endometriosis and cardiovascular diseases, Mu et al. demonstrated a strong connection between endometriosis and hypertension [131]. Endometriosis can be associated with hormonal fluctuations, particularly involving estrogen, which can influence vascular function and potentially contribute to hypertension and to chronic inflammation. Increased concentrations of inflammatory cytokines can promote vasoconstriction and damage to the blood vessels by increased oxidative stress [132].

### 3.3. Experimental Rodent Models of Endometriosis

Research in the field of reproductive disorders, particularly endometriosis, holds significant importance due to its impact on women’s health. One of the earliest models was first reported by Vernon and Wilson [133]. In this model, the uterine horn of a rat was removed, dissected into 2-mm squares, and four of these squares were transplanted into the arterial cascades of the small intestine of another animal from the same species Once implanted, these endometrial fragments establish a local blood supply, respond to hormonal cycling, and develop into cyst-like, vascularized, and innervated lesions, closely mimicking the pathophysiological and pain characteristics of human endometriosis. While this model did not prove effective in investigating the early stages of endometriosis development, it provided valuable insights into endometriosis-associated infertility. This surgically induced model, developed by Vernon and Wilson, remains widely used today as one of the few established methods to mimic endometriosis in rodents. In a recent study, Chauhan et al. applied this method to induce endometriosis lesions in rats and utilized noninvasive photoacoustic imaging to assess various parameters, such as hemoglobin levels, oxygen saturation, and the size of the endometriotic lesions [134]. Notably, they reported the most significant results when the surgical model was combined with the endocrine-disrupting drug diethylstilbestrol (DES). This combination resulted in larger, more complex endometriotic lesions, accompanied by markedly higher inflammatory markers. Chauhan et al. indicate that endocrine disruptors, such as DES, play a pivotal role in the induction of endometriosis. 

Another notable model was developed by Persoons et al. [135], in which the new mouse model developed by Cousins FL and her colleagues was optimized and adapted for use on rats [136]. In ovariectomized rats, high-dose estrogen injections (500 ng/100 µL) were used to mimic the pre-ovulatory surge, followed by low-dose estrogen (5 ng/100 µL) with a progesterone pellet to simulate the implantation window. Decidualization was induced via mechanical uterine stimulation and oil co-injection, and subsequent biochemical and vascular changes were assessed. Persoons et al. reported upregulation of genes involved in prostaglandin synthesis, vasoactive and inflammatory mediators, extracellular matrix remodeling, and leukocyte recruitment. Progesterone withdrawal then triggered menstrual-like shedding of endometrial tissue, which was collected and injected intraperitoneally into recipient rats, resulting in endometriotic lesions. This rat model is also suitable for replicating endometriosis-associated pain, thereby allowing the investigation of targeted therapeutic strategies informed by a deeper understanding of the underlying pathophysiological mechanisms [135]. Table 3 summarizes the rat models of endometriosis.

## 4. Conclusions

In conclusion, this review highlights that gynecological disorders such as POI/POF, PCOS as well as endometriosis are complex conditions in which oxidative stress plays a central role in both reproductive dysfunction and systemic complications, particularly affecting cardiovascular health. Rodent models, especially chemically or pharmacologically induced rat models, remain indispensable tools for investigating the pathophysiological mechanisms underlying these disorders. Our analysis demonstrates that different models offer distinct advantages and limitations, and careful selection is essential to address specific research questions. Moreover, these models provide a valuable platform for evaluating potential therapeutic interventions aimed at mitigating both reproductive and cardiovascular consequences. Taken together, these insights emphasize the need for continued research to refine experimental models, elucidate mechanistic pathways linking oxidative stress to reproductive and systemic dysfunctions, and develop targeted strategies to improve outcomes for affected women.

Our manuscript also has limitations that should be acknowledged. First, we did not include an analysis of genetic models, which may provide additional insights into the mechanistic background of oxidative stress-related pathologies. Moreover, the effects of metabolic disorders across the various experimental models were not examined in detail. Addressing these aspects in future reviews would allow for a more comprehensive understanding of the underlying mechanisms.

## Figures and Tables

**Table 1:** Rat models of POI/POF.

Models	Characteristics	Mechanism of Action	Methods
**Galactose model**	Prohibition of follicular development by targeting primordial/primary follicles during prenatal, and pre-antral/antral follicles during postnatal exposure	Increased GAL-1-P and galactitol levels → Mitochondrial dysfunction Oxidative stress → Granulosa and theca cell damage → Impaired estrogen production and follicle maturation	d-GAL enriched diet (65% food powder/35% d-GAL powder) during different stages of pregnancy [35]
	Limitations	GAL toxicity does not represent common human etiologies	
**Chemotherapy model**	Disruption of the structural and functional integrity of ovarian follicles, leading to dysregulated estrus cycle and E2 production, as well as tissue damage	DNA cross-linking, inhibited DNA transcription and synthesis → Inhibition of vascularization Decreased antioxidant capacity → Induction of follicular apoptosis and necrosis	CIS: 2 mg/kg/day, i.p, for 7 days [37] or 2.5 mg/kg/day, i.p, for 14 days [38] CP: 200 mg/kg, i.p (day 1), then 8 mg/kg, i.p (days 2–14) [40], 50 mg/kg, i.p (day 1), then 8 mg/kg, i.p (days 2–14) [41] CP+BSF: 1× 83.52 mg/kg CP + 1× 20.88 mg/kg BSF, i.p [39]
	Limitations	Ovarian damage depends on drug type, dose, and duration & risk of systemic organ damage	
**Chemically induced model**	Induction of reproductive toxicity through decreased granulosa cell viability	VCD-induced DNA damage and apoptosis → Inhibited cell proliferation	VCD: 80 mg/kg/day, i.p, 15 [42] or 30 days [43]
	Limitations	Ovarian damage depends on drug type, dose, and duration & risk of systemic organ damage	
**Radiation therapy model**	Reduction in ovarian size, as well as altered hormone levels and estrus cycle	Decreased LH, FSH and E2 levels → Dysregulated hormonal signaling	Single pelvic irradiation under CT localization, using either 3.2, 4 or 4.8 Gy doses [44] Single total body irradiation using either 1, 5 or 10 Gy gamma rays delivered by Co60 teletherapy machine [51] Single total body irradiation with 6 Gy gamma rays [52]
	Limitations	Causes severe, irreversible follicular depletion	
**Autoimmune model**	Morphologic changes characterized by irregular follicular shape, as well as general ovarian damage	Decrease in follicles and granulosa cells → Decreased E2 and AMH levels	Ovarian antigen (1:1 ovarian tissue supernatant + FCA/FIA): 3× 0.35 mL, s.c, once every 10 days. Supernatant + FCA (1st immunization), supernatant + FIA (2nd and 3rd immunization) [45] or 4× 0.1 mL, s.c, on the 1st, 14th, 28th and 40th days. Supernatant + FCA (1st immunization), supernatant + FIA (2nd, 3rd and 4th immunization) [53]
	Limitations	Immune activation is often non-selective	
**Chronic stress model**	Hormonal imbalances caused by the disruption of the HPA-axis, as well as histologic changes, including: cortical thickening and structural disorganization of the ovarian stroma, and increased number of atretic follicles	Disregulated HPA-axis signaling → Decreased CRH, ACTH and CORT levels → Altered E2, AMH, GnRH, and FSH levels → Fibrosis	Acousto (65 dB)-optical (3–500 lux, 1/s frequency)–electric (24~36 V) stimulation, 5 times/day at random intervals, for 20 days. Acousto-optical stimulation: 10 s; acousto-optical–electric stimulation: 60 s; Electrical stimulation: 5 s [46] CUMS: daily exposure to a random stress-inducing stimuli (e.g., food and water restriction, wet pads, forced swimming, noise, reversed circadian cycle, etc.) for ≤35 days [47]
	Limitations	Low reproductibility	
**Glycoside model**	Impaired structural integrity, as well as histologic alterations and damage of the ovarian tissue, with subsequent disruption of the estrus cycle and of normal hormonal signaling	Inflammation and oxidative stress → Apoptotic and necrotic cell death → Decrease in primary and secondary folicles → Elevated FSH and LH levels, Decreased E2 and AMH levels	TG: 40 mg/kg/day, per os, for 10 weeks [50], 60 mg/kg/day, per os, for 45 days [54], 75 mg/kg/day, per os, for 14 days [55] or 50 [56] mg/kg/day, per os, for 14 days TWP: 50 mg/kg/day, per os, for 14 days [57]
	Limitations	Ovarian toxicity is poorly defined	

Abbreviations: **GAL-1-P**: galactose-1-phosphate; **d-GAL**: d-galactose; **CIS**: cisplatin; **CP**: cyclophosphamide; **BSF**: busulfan; **VCD**: 4-vinylcyclohexene dioxide; **FCA**: Freund’s complete adjuvant; **FIA**: Freund’s incomplete adjuvant; **HPA-axis**: hypothalamic–pituitary–adrenal axis; **CUMS**: chronic unpredictable mild stress; **TG**: Tripterygium glycoside; **TWP**: Tripterygium wilfordii polyglycoside.

**Table 2:** Rat models of PCOS.

Model	Characteristics	Mechanisms of Action	Methods
**DHEA-** **induced model**	Reproduces key human PCOS features (cysts, anovulation, hormonal imbalance)	Elevated androgens suppress aromatase → Decrease in 2 production and impaired follicular maturation	Pre-pubertal rats received s.c DHEA injection (30 mg/kg) for 12 weeks [87]; Post-pubertal rats received s.c. DHEA injection (60 mg/kg) for 20–30 days [88]
	Limitations	Often lacks metabolic phenotype; reproducibility is protocol-sensitive	
**DHT-** **induced model**	Applicable to prenatal/postnatal rats without estrogen conversion effects	Non-aromatizable androgen → cannot convert to E2 → persistent hyperandrogenism	Prenatal: DHT 3 mg/day, s.c, on the 16th to 19th gestational day (GD16–19). Postnatal: 7.5 mg/pellet implanted, 90-day release [102]; Postnatal: 5 mg/kg/day, s.c, for 7 days [89]
	Limitations	Highly timing/dose-dependent; ovarian size may be reduced	
**Letrozole-** **induced model**	Irregular estrous cycles, cystic ovarian morphology, increased LH levels	Aromatase inhibition blocks androgen–estrogen conversion → hyperandrogenism	1 mg/kg orally ~21 days [103]; 0.5 mg/kg, per os, for 21 days [104]
	Limitations	Estrogen-block model; not fully representative of human hyperandrogenic PCOS; metabolic signs often absent	
**Testosterone-** **induced model**	Irregular estrous cycles and increased preantral/antral follicles	Prenatal testosterone disrupts the HPO axis → altered ovarian morphology and hormonal profile in adulthood	Pregnant rats injected (s.c) with 5 mg free testosterone on gestational day 20 [97]. Postnatal: 21-day-old rats received daily testosterone propionate s.c ~35 days (1 mg/100 g) [105]
	Limitations	Weak metabolic phenotype; strong dose/age dependency	
**Progesterone receptor** **antagonist (RU486) model**	Follicular growth arrest, increased follicular atresia, increased serum LH	Progesterone receptor antagonism inhibits follicle development, ovulation, and corpus luteum formation	Often postnatal treatment; 4 mg/body/day RU486/mifepristone with an osmotic mini-pump ~2 weeks [99]
	Limitations	Weak androgen/metabolic features; highly dose-dependent phenotype	
**Estradiol valerate (EV)-induced model**	Cystic ovarian morphology; high LH/FSH ratio, disrupting follicle maturation	Prolonged high-estrogen state suppresses FSH and alters GnRH → increase in LH/FSH ratio → follicular arrest & hyperandrogenism	Young rats injected (i.m) with EV (2 mg dose) [101]. Or received a single i.m. injection of 5 mg EV ~32 days [106]
	Limitations	Estrogen-dominant; lacks metabolic/endocrine PCOS traits; ovarian weight reduction	

**Table 3:** Rat modes of endometriosis.

Model	Characteristics	Mechanisms of Action	Methods
**Uterine horn model**	Forms vascularized, innervated cyst-like lesions mimicking human endometriosis and pain symptoms	Establishes local blood supply and responds to hormonal cycling	Uterine horn tissue (~2 mm) transplanted from donor to recipient into the small intestine [133] + DES [134]
	Limitations	Highly invasive & non-physiological; does not mimic the natural menstrual cycle; no early disease stage	
**Menstruating rat endometriosis model**	Mimics menstrual cycle and pain; suitable for testing therapies	Hormonal induction causes decidualization and menstrual-like shedding → lesion formation	OVX rats; 500 then 5 ng/100 µL estrogen + progesterone; mechanical decidualization; menstrual tissue injected into recipient, i.p. [135]
	Limitations	Invasive; difficult to reproduce due to complex hormone dosing; artificial cycle	

## Abbreviations

The following abbreviations are used in this manuscript:

AMH	Anti-Müllerian Hormone
E2	Estradiol
FSH	Follicle-Stimulating Hormone
GnRH	Gonadotropin Releasing Hormone
LH	Luteinizing Hormone
PCOS	Polycystic Ovary Syndrome
POF	Premature Ovarian Failure
POI	Primary Ovarian Insufficiency
ROS	Reactive Oxygen Species
